# Rotavirus Vaccination of Premature Newborns in the NICU: Evaluation of Vaccination Rates and Safety Based on a Single-Centre Study

**DOI:** 10.3390/vaccines11081282

**Published:** 2023-07-26

**Authors:** Klaudia Marcinek, Paweł Zapolnik, Renata Radziszewska, Agnieszka Ochoda-Mazur, Hanna Czajka, Dorota Pawlik

**Affiliations:** 1 Neonatology Clinical Department, University Hospital in Kraków, 31-501 Kraków, Poland; 2 College of Medical Sciences, University of Rzeszów, 35-315 Rzeszów, Poland; 3 Medical College, Jagiellonian University, 31-008 Kraków, Poland

**Keywords:** rotavirus, vaccines, new-born, neonatology, neonatal intensive care unit, vaccinology, COVID-19

## Abstract

Preterm newborns are babies born before the end of the 36th week of gestational life. They are at increased risk of infection and death from infectious diseases. This is due, among other things, to the immaturity of the immune system and the long hospitalisation period. One common infectious disease in the paediatric population is rotavirus (RV) infection. We now have specific vaccines against this pathogen. The aim of this study was to evaluate the safety of rotavirus vaccination in the neonatal intensive care unit (NICU) setting and to determine the tolerance of this vaccine in low- and extremely low-weight children. The study carried out at a single centre, the University Hospital in Kraków, also allowed the assessment of vaccination trends during the severe acute respiratory syndrome coronavirus 2 (SARS-CoV-2) pandemic. During the observation period, 126 premature newborns received the RV vaccine. We observed no adverse effects, and our analysis shows safety and good tolerance of the vaccine among preterm babies. In addition, we observed an increase in vaccination rates between 2019 and 2021, partly explained by parents’ anxiety about infectious diseases in the era of pandemics and partly explained by a change in vaccination policy in Poland and the introduction of refunding for RV vaccination.

## 1. Introduction

Newborns born prematurely are babies born before the end of the 36th week of gestational life (36 weeks and 6 days). According to world statistics, more than 10% of babies are born prematurely; in Poland, the percentage is >6%. From this group, approximately 70% are born in the 34th week of gestation or later, and the remaining are born before the 34th week of pregnancy. Preterm infants have an increased risk of infection and death from infectious diseases. This is due, among other things, to the immaturity of the immune system and the shorter time for antibodies to cross the placenta from the mother. This process starts at the 17th week of pregnancy and is most active after the 32nd week. An additional factor that increases the risk of infection is a long period of hospitalisation [1,2,3] and exposure to invasive medical interventions.

Many infectious diseases can be prevented via vaccination. Scientific societies recommend vaccination according to the vaccination programme for preterm infants in stable clinical conditions after correct qualification. To qualify for vaccination, a preterm baby must have a stable clinical condition regarding respiratory and circulatory function. Active infection and metabolic diseases must be excluded, as well as progressive renal failure and neurological disorders. Weight gain is also an important parameter [1,4].

One of the available vaccines used in neonatal intensive care units is a vaccine against rotavirus infection [5]. Rotavirus (RV) is a species belonging to the *Reoviridae* family, characterised by the absence of an envelope and genetic material in the form of a double-stranded ribonucleic acid (dsRNA) molecule. The diameter of a single virion is 100 nm. It consists of a three-concentric protein shells, surrounding the dsRNA genome. Apart from this, the virus also contains an viral RNA polymerase and a capping enzyme. It was first detected and identified as the aetiological agent of diarrhoea in 1973 [6,7]. It is the most common agent of gastrointestinal infections in children of <5 years of age and the leading cause of hospitalisation for diarrhoea [5,8]. Newborns born prematurely with a body weight of <2500 g have a 1.6–2.8-fold higher risk of hospitalisation for rotavirus infection in the first year of life compared to children born on time [9].

Two live rotavirus vaccines recommended by the World Health Organisation (WHO) are currently available worldwide. Both products, RotaTeq (Merck & Co., West Point, PA, USA) and Rotarix (GlaxoSmithKline Biologicals, Rixensart, Belgium), are licensed in more than 100 countries. They have an oral form of administration. The microorganisms used for the vaccines are live attenuated variants of naturally occurring strains. The vaccines were obtained after multiple rounds of passage in cell culture. Currently, two available vaccines are a monovalent vaccine using an attenuated strain of G1P[8] (Rotarix) and a pentavalent human–bovine reassortant vaccine (RotaTeq). A tetravalent rhesus monkey–human reassortant vaccine (RRV-TV, RotaShield, Wyeth Laboratories, Marietta, PA, USA) was also available in the past. However, it was withdrawn from the market due to an increased risk of intussusception. The overall efficacy of both vaccines reaches 85% but varies depending on the vaccinated population, child mortality in a specific geographical area or the wealth level of the country. There are other vaccines available in some areas of the world that have passed clinical trials and received approval from several countries. Examples include the Lanzhou lamb Rotavirus vaccine based on the G10P[12] genotype (LLR, Lanzhou Institute of Biological Products, Gansu, China) and the Rotavin-M1 vaccine (POLYVAC-Vietnam, Hanoi, Vietnam) based on the same attenuated G1P[8] strain as the Rotarix vaccine. Clinical trials are currently being conducted to implement new live attenuated rotavirus vaccines [10,11].

The Polish Society of Vaccinology recommends vaccination in children in the optimal clinical condition and time frame, which for extremely preterm infants would be the period of hospitalisation [5]. Vaccination eligibility may, however, be limited due to concerns about the safety of the vaccine regarding the possible risk of transmission of vaccine viruses to other patients in neonatal intensive care units (NICUs).

In the present study, we provide an analysis of the implementation of vaccination against rotavirus infections in a group of premature infants hospitalised in the Neonatology Clinical Department of the University Hospital in Kraków between 2019 and 2021. The analysis was undertaken to answer whether or not vaccination against this disease can be safely performed in a hospital setting and what the tolerance to administering this vaccine in a group of children with low and extremely low body weight is. The study was initiated before the introduction of mandatory vaccination against rotavirus infection in Poland (2021) [1] and continued after the change in the vaccination programme. An additional focus of the evaluation was the analysis of information collected in telephone interviews from parents of previously vaccinated children about the continuation of vaccination against rotavirus infections in these children during the severe acute respiratory syndrome coronavirus 2 (SARS-CoV-2) pandemic.

## 2. Materials and Methods

### 2.1. Methods

Neonates born prematurely (<36 completed weeks of gestation) in the Neonatology Clinical Department of the University Hospital in Kraków were included in the study. The inclusion criterion was the period of hospitalisation in the department for a minimum of 42 days. The primary evaluation parameter was the vaccination coverage and safety of RV vaccination when it was not mandatory—that is, non-refundable (2019–2020; monovalent vaccine Rotarix, GlaxoSmithKline Biologicals, Rixensart, Belgium). Another comparative parameter was vaccination coverage following the introduction of the refundable RV vaccine (2021; five-valent vaccine RotaTeq, Merck & Co., West Point, PA, USA). The parameters assessed were body weight, Apgar score at 5 min, length of hospitalisation, total parenteral nutrition (TPN) time, day of administration of the first vaccine dose and weight of the child on the day of vaccination. After discharge from the hospital (when the patients were 8–12 months old), a questionnaire was conducted with the parents asking them questions about the continuation of vaccination, the occurrence of diarrhoea, its diagnosis and the possible need for hospitalisation, and the child’s current health status. In addition, due to the development of the coronavirus disease 2019 (COVID-19) pandemic, we also carried out an analysis of vaccination rates in the subsequent years of the pandemic. The study received a positive opinion from the Bioethics Committee of Jagiellonian University (no. 1072.6120.220.2020 of 24 September 2020).

### 2.2. Statistical Analysis

Variables were described using the following statistics: mean +/− standard deviation, median and range (minimum and maximum values). Categorised data were presented as counts and percentages. The significance of differences in gestational week groups for quantitative data was verified via the Kruskal–Wallis test and qualitative data were verified via Pearson’s chi-square test. Differences between vaccinated and unvaccinated groups were tested with the Mann–Whitney U-test. Calculations were performed using Statistica 13.1. software (StatSoft Poland, Kraków, Poland). A statistical significance level of *p* < 0.05 was applied.

## 3. Results

In total, 126 preterm infants received the rotavirus vaccine during the observation period. Most (N = 68) were in the 29–32-week gestation age group and the smallest number of infants were (N = 5) in the 33–36-week gestation group. Significant differences in birth weights, hospitalisation length, and TPN time were observed. The baseline characteristics of the study group are shown in Table 1.

According to Polish guidelines and the recommendations of the European Society for Paediatric Infectious Diseases (ESPID) [5,12], the first dose of the vaccine was administered after the patient had reached 6 weeks of age (42 days) until the end of 12 weeks of age (84 days). All newborns at the time of vaccination remained in good general condition allowing discharge home or stable for their primary medical condition (prematurity). They were starting to learn to feed with a teat, receiving treatment for retinopathy of prematurity (ROP), remained on self-breathing or required high-flow nasal cannula (HFNC) respiratory support or passive oxygen therapy. In addition, all children had reached total feeding tolerance at the time of vaccination. After vaccination, children were observed in the department for 1–2 days (76 children—60%), for 3–7 days (44 children—35%), or longer periods (6 children—5%). The analysis of the eligibility for the vaccination in the NICU is presented in Table 2.

The reasons for not vaccinating against RV infections during the hospitalisation period in the NICU were (1) a lack of parental consent, (2) congenital cytomegalovirus infection, (3) very massive inguinal hernias, (4) recurrent infections making vaccination impossible and (5) severe, persistent abdominal pain.

In 2019–2020, rotavirus vaccination was optional (not refunded), and therefore, not all parents wanted to cover the cost. Two parents refused to vaccinate their children in 2021 (after the introduction of vaccine refunding). We defined ‘severe, persistent abdominal pain’ as any Neonatal Pain, Agitation and Sedation Scale (NPASS) score of +1 or +2 on physical examination of the abdomen.

It is noteworthy that one child, after vaccination in the primary department, was transferred to another centre for the treatment of retinopathy of prematurity, where a stool test routinely performed on admission confirmed the presence of rotavirus. At the same time, the patient had no gastrointestinal symptoms. With large-scale rotavirus vaccination, such events may occur, and therefore the procedures for reporting epidemic outbreaks should not be used in this case.

After discharge from the hospital, we conducted a questionnaire survey among parents. Ultimately, 105 questionnaires were successfully analysed. According to the interviewed parents, the vast majority (85%) of the children developed normally following their adjusted age. Severe complications of prematurity in the form of neurological disorders and developmental delays occurred in six children. One child, with intraventricular haemorrhage (IVH) detected during a biophysical profile test (BPP), died in the 13th month of life due to severe liver damage (condition after grade III IVH, cholestasis, and bronchopulmonary dysplasia). Two children vaccinated in the department in all years had diarrhoea due to rotavirus, but none required hospitalisation. None of the parents reported an adverse event following immunisation (AEFI) through the primary care doctor after these vaccinations.

A summary of the group of preterm infants vaccinated compared to all those hospitalised between 2019 and 2021 in the Neonatology Clinical Department of the University Hospital in Kraków is presented in Table 3 and Table 4.

## 4. Discussion

In 2021, the rotavirus vaccine was used in 118 countries [13]. However, vaccination rates (especially in developing countries) and approaches to the possible timing of the first dose vary [14]. The Polish Society of Vaccinology recommends administering the first dose of the vaccine after the age of 6 weeks until the age of 12 weeks. The German and ESPID recommendations are the equivalent [5,12,15]. The United Kingdom (UK) vaccination programme favours the administration of Rotarix (the only vaccine available in the UK) between 8 and 12 weeks of age, with the possibility of application after 6 weeks of age up to a maximum of 14 weeks and 6 days of age [16]. Australian recommendations indicate the possibility of vaccination to be after 6 weeks of age before 12 weeks of age (RotaTeq), or 15 weeks of age (Rotarix) [17]. In contrast, the United States (US) recommendations prepared by the Advisory Committee on Immunization Practices (ACIP) set the maximum time for the first dose to be given at 14 weeks and 6 days of life and further specify that vaccination should be administered on the day of discharge from hospital, due to the theoretical risk of transmission of the attenuated strain to other children [18].

A fundamental aspect related to the use of vaccination against rotavirus infection, and vaccination in general, is its safety. In randomised trials [8,19,20], no increased incidence of apnoea or other respiratory or circulatory disorders was observed after vaccination compared to the placebo group. In a few cases out of 100,000 vaccinated children, intestinal intussusception occurred and was associated with vaccination (especially in the first seven days). Still, the significant benefits of vaccination outweigh the risk of possible adverse events [21,22,23,24]. Furthermore, observational studies and systems collecting information on adverse events have not reported worrying reports after the rotavirus vaccine [25,26,27]. In our study, we also reported no adverse reactions during the observation period of the children in the hospital. A follow-up analysis using questionnaires conducted at the end of the patients’ first year of life also showed no reports of adverse vaccine reactions. Despite the smaller study group, our analysis shows safety and good tolerance of the vaccine among preterm infants, which aligns with the results of larger studies on the safety of rotavirus vaccination.

Another aspect worth mentioning is the risk of transmission of vaccine viruses between patients in the neonatal intensive care unit. Antigens or the RNA of attenuated viruses can be detected in the stools of vaccinated babies over a certain time. The excretion of replication-competent virions has been found in approximately 16% of infants born at term [28,29]. Theoretically, there is a risk of virus transmission to other patients, but to date, no symptomatic infections with vaccine strains have been reported in hospital wards following standard hygiene procedures [30,31,32,33]. Our observations confirm the quoted results. During the follow-up of patients after vaccination, no symptomatic gastrointestinal infection of rotavirus aetiology was found in other newborns. However, isolated cases of transmission of the vaccine virus to other children (siblings) in out-of-hospital (home) conditions, where medical hygiene procedures are usually not maintained, have been reported in the literature [34,35,36]. Analysing the possibility of virus transmission in the out-of-hospital setting was not the aim of our study, so we cannot directly refer to the above reports.

Slightly different but truly interesting results of an analysis of vaccination rates were obtained by van Dongen et al. [37]. The authors conducted a cohort study in 13 Dutch hospitals, administering RV vaccination to newborns born prematurely, with low birth weights, and with congenital defects. Ultimately, more than 700 patients were vaccinated. Unexpectedly, there was limited vaccine efficacy and 20 cases of severe rotavirus-associated gastrointestinal infection among the vaccinated children, of which 2 required hospitalisation. It is possible that this effect was caused by the immaturity of the immune system of preterm infants or the different microbiota of these patients, which may also influence the generation of vaccine responses [38]. The impact of the epidemiological characteristics of the Netherlands, where vaccination against rotavirus infection is not part of the immunisation programme and vaccination rates in the private sector are at a low level, cannot be excluded either [24,37].

However, it is worth pointing out that the authors also analysed an additional factor—whether or not children received RV vaccines simultaneously with other vaccinations according to the national vaccination programme or in a 3-day interval. Vaccination was generally well-tolerated, but with coadministration, the risk of gastrointestinal adverse events increased by about 10 per cent [37]. In our research, we focused exclusively on RV vaccination without assessing coadministration. In addition, the different nature of the study and the population makes it difficult to directly compare the results of the two analyses, despite the fact that we did not record any adverse reactions after vaccination.

Costantino et al. [39] conducted an observational, descriptive, non-controlled, and non-randomised study in six public hospitals with NICUs. They evaluated the safety of a monovalent rotavirus vaccine (Rotarix) in a group of preterm infants. Vaccinations were performed in newborns born at or after 28 weeks of gestation. Children with unstable clinical conditions, previous episodes of intussusception, necrotising enterocolitis (NEC), gastrointestinal defects and immunodeficiencies were excluded from the study. However, 449 newborns were included in the study. The gender structure of the study group was similar to that in our study in 2019, with 223 male (49.7%) and 226 female (50.3%) newborns. The mean body weight on the day of the first dose of the vaccine was 3388.5 g (with a standard deviation of 903.4 g), which does not correlate with our results. However, in the cited study, the most significant number of newborns were vaccinated at 33 weeks of gestation, in contrast to that in our study, where the largest group of vaccinated infants was of newborns at 29–32 weeks of gestation. Only a tiny percentage (0.2%) of vaccinated neonates experienced adverse events. These were mainly diarrhoea, colic abdominal pain and fever of > 38.5 degrees Celsius. The above symptoms were self-limiting and subsided within a short period of 24 to 48 h. These results, like ours, support the safety of rotavirus vaccinations in preterm infants in NICU conditions. Our study used monovalent and polyvalent rotavirus vaccines because of the Polish health policy and vaccine availability. Costantino et al. [39] used only monovalent vaccines in their trial. Another difference in this study from our analysis was that only 8.1% of vaccinations were initiated in the NICU. In the remaining cases, vaccination was performed in outpatient centres, which we did not take into consideration in our study.

Finally, it is also worth citing the study by Yoon et al. [40], who presented a different analysis to that of our trial. The authors analysed 431 faecal samples from neonates hospitalised in the NICU at a single centre in South Korea. Patients were divided into those born in that hospital (123 neonates) and those admitted from outside (287 neonates). Ultimately, 410 children underwent RV antigen testing, with a positive result in 180 cases. Statistically, infection was significantly more frequent in babies with a birth weight of less than 2500 g, postmenstrual age (PMA) of less than 37 weeks of gestation, and those born by caesarean section, confirming previous observations. In addition, the research team conducted a viral genotyping analysis, identifying the genotype in 131 samples, the absolute majority of which were of the G4P[6] genotype (95.4%). The authors did not analyse vaccination rates in a single centre, as we did, but they focused on an epidemiological assessment of the prevalence of rotavirus infection in the NICU patient population over a five-year period following the introduction of rotavirus vaccination in South Korea. While we cannot directly compare the results, it is worth noting that the introduction of population-based vaccination will not fully protect preterm infants from subsequent rotavirus infections, making it worth highlighting the relevance of vaccination conducted in the NICU [40].

The interesting data obtained in the above study and the incomplete knowledge of vaccination against rotavirus infection emphasise the need for further studies evaluating the efficacy and safety of these vaccines. Knowledge on this subject should also be supplemented by the safety aspects of administering different vaccines simultaneously. The COVID-19 pandemic significantly impacted the epidemiology of other infections and vaccination rates. In our study, vaccination rates in the NICU increased during the pandemic’s evolution, but this coincided with the introduction of RV vaccination as refundable. Therefore, it is difficult to assess whether or not the increase in vaccines applications is a matter of parents’ anxiety about infectious diseases in the pandemic era or rather a change in vaccination policy in Poland. In conclusion, despite the widespread use of RV vaccination in many countries, we still do not have accurate data on its use among preterm infants and its potential benefits and risks. This area of vaccinological knowledge requires further research and a constant updating of recommendations.

## Figures and Tables

**Table 1:** Characteristics of the rotavirus vaccinated population of preterm newborns. Values shown include a median and a range of minimum and maximum values and a mean with standard deviation.

Feature	24–28 PMA	29–32 PMA	33–36 PMA	*p*-Value
N	53	68	5	
Birth weight (g)	980 [500–1400] 926.83 ± 230.3	1330 [660–2095] 1343.53 ± 317.84	1770 [1270–2150] 1762 ± 330.33	0.0000
Apgar score 5 min	7 [4–9]	8 [3–9]	9 [6–9]	-
Length of hospitalisation (days)	98.5 [56–221] 106.4 ± 37.9	63.5 [45–157] 67.03 ± 20.44	47 [45–74] 53.2 ± 11.69	0.0000
Total parenteral nutrition (days)	27 [11–58] 28.04 ± 12.65	11 [0–60] 14.88 ± 10.74	6 [5–16] 8.25 ± 5.19	0.0000

Abbreviations: PMA—post menstrual age.

**Table 2:** Vaccinations performed according to gestational age, body weight and days in the Neonatal Intensive Care Unit. Values shown include a median and a range of minimum and maximum values and a mean with standard deviation.

	24–28 PMA	29–32 PMA	33–36 PMA	*p*-Value
Day of 1st dose in NICU	72 [46–90] 70.06 ± 10.61	57 [43–94] 14.88 ± 10.74	48 [45–67] 50.8 ± 9.23	0.0000
Body weight (g) (1st dose in NICU)	2400 [1680–3280] 2425.56 ± 448.13	2690 [1900–4720] 2767.31 ± 479.96	3680 [2380–3850] 3260 ± 685.53	0.0007
Number of babies vaccinated with 2nd dose	18	2	0	-
Day of 2nd dose in NICU	118 [84–144] 110.95 ± 30.75	114 [101–127] 114 ± 18.38	-	-
Body weight (g) (2nd dose in NICU)	3340 [2580–4490] 3381.33 ± 539.48	3130 [3130–3130]	-	-
Number of babies vaccinated with 3rd dose	4	1	0	-
Day of 3rd dose in NICU	163,5 [135–186] 123.5 ± 83.11	157	-	-
Body weight (g) (3rd dose in NICU)	3990 [3580–4400] 3990 ± 410	3540	-	-
Vaccination after discharge (continuation)	28 (52.8%)	55 (80.9%)	4 (80%)	0.7469

Abbreviations: NICU—Neonatal Intensive Care Unit; PMA—post menstrual age.

**Table 3:** Number of study participants (vaccinated and unvaccinated) at each time point in 2019–2021. ‘Vaccinated’—vaccinated patients; ‘Unvaccinated’—unvaccinated patients who could have been vaccinated. Stay in NICU up to 77 days—high chance of vaccination; stay in NICU up to 84 days—little chance of vaccination after 77 day; stay in NICU up to 84 days—no chance of vaccination. Values are shown as total numbers.

Feature	2019		2020		2021	
Vaccination Status	Vaccinated	Unvaccinated	Vaccinated	Unvaccinated	Vaccinated	Unvaccinated
Stay in NICU up to 77 days	1	61	37	26	40	12
Stay in NICU up to 84 days	2	7	2	2	2	1
Stay in NICU for more than 84 days	9	8	15	7	20	10
Total	12	76	54	35	62	23
Total number of study participants	88		89		85	

Abbreviations: NICU—Neonatal Intensive Care Unit.

**Table 4:** Premature newborns vaccinated against rotavirus compared to all infants born prematurely in 2019–2021. ‘Vaccinated’—vaccinated patients; ‘Unvaccinated’—unvaccinated patients who could have been vaccinated. Values are shown as total numbers and percentages. The percentage of children vaccinated increased yearly.

Feature	2019	2020	2021
Total of preterm newborns in NICU	442	432	353
Gender of the preterm newborns—female/male	50%/50%	64%/36%	46%/54%
Number of study participants in relation to all NICU patients	88/442 (19.91%)	89/432 (20.60%)	85/353 (24.08%)
Number of vaccinated newborns in relation to all NICU patients	12/442 (2.71%)	54/432 (12.5%)	62/353 (17.56%)
Number of vaccinated newborns in relation to all study participants (vaccinated + unvaccinated)	12/88 (13.64%)	54/89 (60.67%)	62/85 (72.94%)

Abbreviations: NICU—Neonatal Intensive Care Unit.

## Data Availability

The data presented in this study are available on request from the corresponding author. The data are not publicly available due to the limited number of patients and the single-centre approach of the study.

## Abbreviations

ACIP	Advisory Committee on Immunization Practices
AEFI	Adverse event following immunisation
BPP	Biophysical profile test
COVID-19	Coronavirus disease 2019
ESPID	European Society for Paediatric Infectious Diseases
HFNC	High-flow nasal cannula
IVH	Intraventricular haemorrhage
NEC	Necrotising enterocolitis
NICU	Neonatal Intensive Care Unit
NPASS	Neonatal Pain, Agitation and Sedation Scale
RNA	Ribonucleic acid
ROP	Retinopathy of prematurity
RV	Rotavirus
SARS-CoV-2	Severe acute respiratory syndrome coronavirus 2
TPN	Total parenteral nutrition
WHO	World Health Organisation
